# Transcription modulation of pathogenic streptococcal and enterococcal species using CRISPRi technology

**DOI:** 10.1371/journal.ppat.1012520

**Published:** 2024-09-19

**Authors:** Natalie G. Alexander, William D. Cutts, Thomas A. Hooven, Brandon J. Kim

**Affiliations:** 1 Department of Biological Sciences, University of Alabama, Tuscaloosa, Alabama, United States of America; 2 Department of Pediatrics, University of Pittsburgh School of Medicine, Pittsburgh, Pennsylvania, United States of America; 3 Richard King Mellon Institute for Pediatric Research, University of Pittsburgh Medical Center, Pittsburgh, Pennsylvania, United States of America; 4 UPMC Children’s Hospital of Pittsburgh, Pittsburgh, Pennsylvania, United States of America; 5 Department of Microbiology, Heersink School of Medicine, University of Alabama at Birmingham, Birmingham, Alabama, United States of America; 6 Center for Convergent Biosciences and Medicine, University of Alabama, Tuscaloosa, Alabama, United States of America; 7 Alabama Life Research Institute, University of Alabama, Tuscaloosa, Alabama, United States of America; University of Geneva: Universite de Geneve, SWITZERLAND

CRISPR, short for clustered regularly interspaced short palindromic repeats, is found in 40% of bacteria and 85% of archaea providing defense against phage and other foreign DNA [1–3]. The CRISPR locus contains both “spacers,” which encode guide RNAs (gRNAs) used to direct the system to target nucleic acids, and Cas effector proteins. There are several Cas effector proteins with some functioning as endonucleases, while others serve for recognition or processing of gRNA. Certain Cas effectors, such as Cas9, can be directed to cut a given site via recognition of a gRNA, creating a double-stranded break in the target DNA. These effectors can scan long sequences of DNA for short sequences called protospacer adjacent motifs (PAMs), before unraveling the DNA to cut. These PAMs vary between different CRISPR-Cas types [4,5].

CRISPR interference (CRISPRi) utilizes a catalytically dead Cas effector (dCas), through targeted mutation of the catalytic domains, specifically HNH and RuvC-like nuclease domains in Cas9, to eliminate Cas endonuclease activity [4,5]. This dCas effector retains its ability to recognize a gRNA and bind DNA. If guided to a gene, dCas will bind and form a “roadblock” on the gene, reducing transcription and causing a knockdown in expression of the gene as shown in Fig 1.

**Fig 1:**
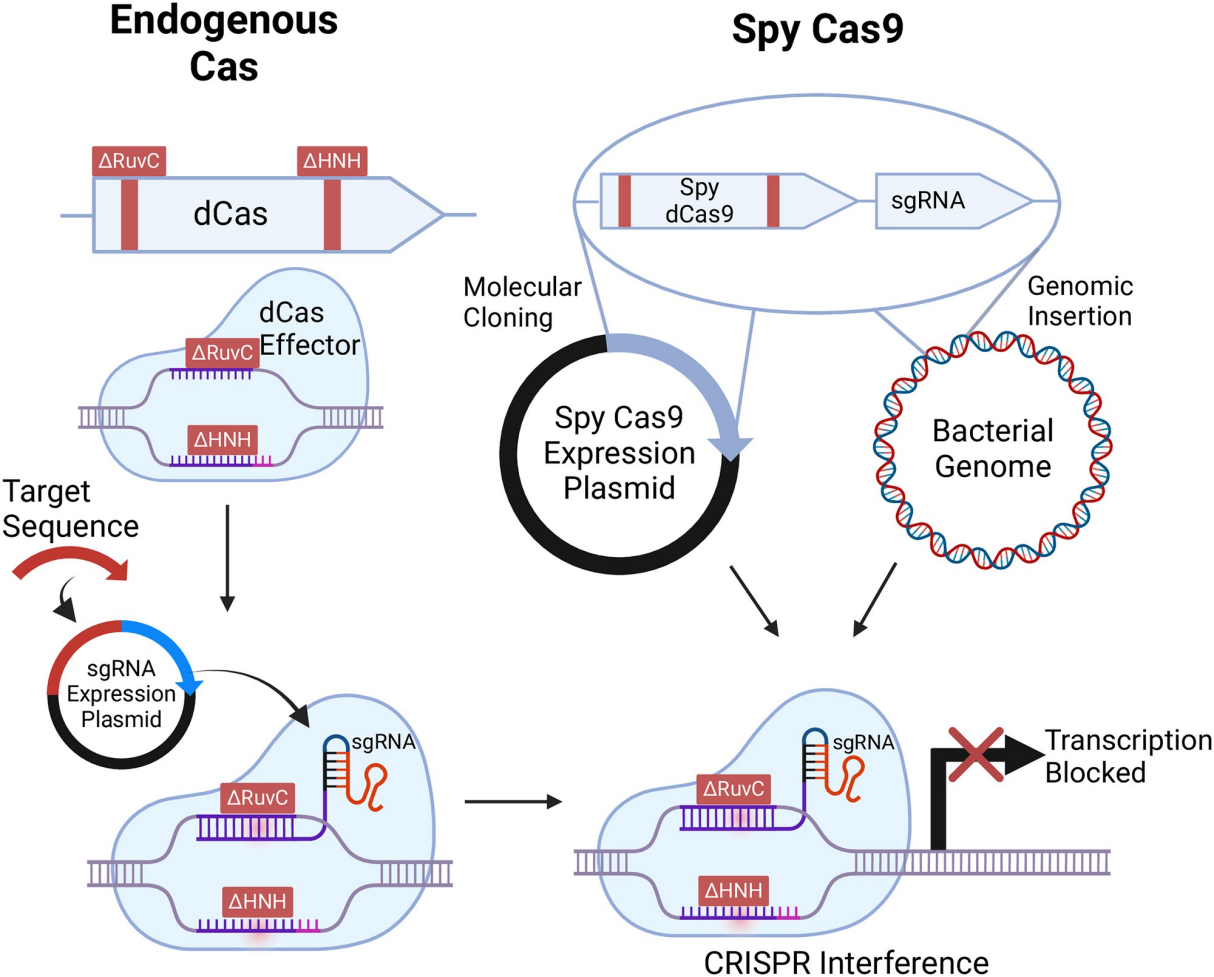
CRISPRi can be approached in a species-dependent manner, either utilizing an organism’s endogenous Cas effector protein or via the introduction of Spy dCas9. Either of these approaches entails targeted mutagenesis of the HNH and RuvC-like nuclease domains responsible for Cas9’s cutting activity. If this catalytically dead Cas9 is endogenous, a plasmid can be transformed containing a protospacer that is expressed as a sgRNA targeted to a gene of interest to produce a knockdown. If using Spy dCas9 non-endogenously, the gene will either be chromosomally inserted into your target organism or expressed on a plasmid with the corresponding targeting protospacer. Fig 1 created in BioRender. CRISPR, clustered regularly interspaced short palindromic repeats; CRISPRi, CRISPR interference; dCas, dead Cas; sgRNA, single-guide RNA; Spy dCas9, *S*. *pyogenes* dCas9.

This can be most easily achieved in Class 2 Cas effectors (primarily Cas9 and Cas12a), as these rely primarily on a single effector for both target binding and DNA cleavage rather than the effector complexes of Class 1 systems, which feature a cascade of Cas proteins (shown in Fig 2) [6,7]. dCas transcriptional regulators allow for near-silencing of multiple genes making CRISPRi technology robust and high throughput. CRISPRi systems can be modified and applied to a variety of bacteria.

**Fig 2:**
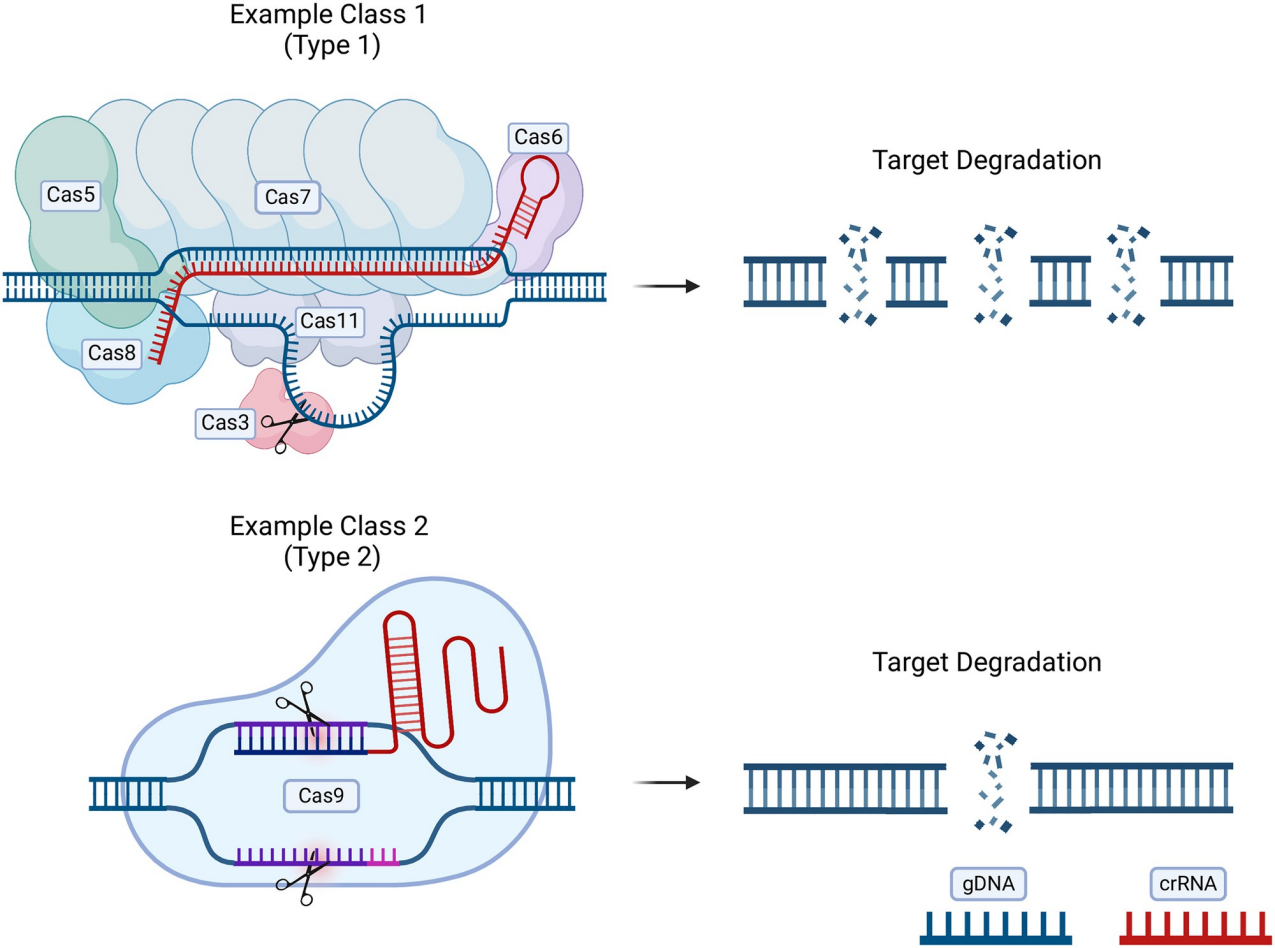
Class 1 and 2 CRISPR-Cas systems vary significantly, with class 2 systems being more user-friendly for CRISPRi. Class 1 systems involve a cascade with a number of Cas proteins being utilized to achieve target recognition, binding, and targeted cleavage. Class 2 systems utilize a single effector such as Cas9 or Cas12 for target recognition, binding, and targeted cleavage. Fig 2 created in BioRender. CRISPR, clustered regularly interspaced short palindromic repeats; CRISPRi, CRISPR interference; crRNA, CRISPR RNA; gDNA, genomic DNA.

We will review recent examples of CRISPRi technology in *Enterococcus* spp., *Streptococcus pyogenes*, *Streptococcus agalactiae*, and *Streptococcus pneumoniae*. We will also discuss future applications of CRISPRi technology including direct genome editing, gene silencing, and CRISPRseq.

## *Enterococcus* spp.

*Enterococcus faecalis* is a gram-positive opportunistic pathogen known to cause multidrug-resistant infections including sepsis, endontic infections, and hospital-acquired urinary tract infections [8–10]. *E*. *faecalis* possess a type-II CRISPR-Cas9 system. Interestingly, *E*. *faecalis* clinical isolates that are multidrug-resistant have mobile genetic elements that can be targeted by CRISPR-Cas systems and then maintained [11]. *E*. *faecalis* has shown that there is a phenotype of “CRISPR tolerance,” which corresponds to transient maintenance of targeted DNA [11].

Targeted mutagenesis through allelic exchange has been a frequently used method for genome editing. An alternative to this method was developed in *E*. *faecalis*, which utilizes a two-plasmid CRISPR-Cas9-mediated genome editing system that allows for a reduction of screening needed, as well as a shortened period for genome editing [12]. Another two-plasmid system was generated in *Enterococcus faecium*, utilizing inducible expression of RecT, an ssDNA-annealing protein associated with phage recombination, in combination with CRISPR-Cas9-based counterselection. This system permits either genomic insertion or deletion while minimizing screening, making it a rather versatile approach to mutagenesis [13]. Limitations of this system include reduced transformation efficiency due to the toxicity of Cas9, as well as the need for multiple large vectors [14].

An *E*. *faecalis* CRISPRi tool was created for use in high-throughput, scalable genetic control studies eliminating the limitation around reduced transformation results with the genome editing system. A dual-vector nisin-inducible system was tested and shown to silence both single genes and whole operons [15]. Additionally, the system was able to silence genes involved in biofilm formation and antibiotic resistance [15]. Future applications include investigating antibiotic resistance mechanisms and virulence factors associated with *E*. *faecalis* infection.

## Streptococcus pyogenes

*S*. *pyogenes* is a gram-positive bacterium responsible for many infections including pharyngitis, necrotizing fasciitis, and toxic shock syndrome [16–18]. *S*. *pyogenes* can possess either CRISPR Type I-C or Type II-A.

*S*. *pyogenes* is notable for the Cas9 system known as Spy Cas9, which was utilized to develop CRISPRi [19]. The process entailed cloning the *S*. *pyogenes* Cas9 gene along with a minimal CRISPR locus into either an expression vector or the target organism’s genome. Spy Cas9 has been used extensively as a platform for producing CRISPRi functionality in bacteria that do not harbor an endogenous Cas9 [5,20].

While Spy Cas9 has been used to apply CRISPRi in other species, the same could not be said for *S*. *pyogenes* until recently. A study has shown successful implementation of a doxycycline-inducible dCas9-based CRISPRi system in *S*. *pyogenes* [21]. The doxycycline induction system permits tight expression regulation, and researchers were able to demonstrate that the system could reliably knock down target genes of interest. A library of unique spacers featuring near-complete coverage of the *S*. *pyogenes* genome was made accessible in SpyBrowse, the *S*. *pyogenes* genome browser. Future directions include the transformation and implementation of the spacers, and generation of a CRISPRi knockdown library.

Additionally, it has been demonstrated that in *S*. *pyogenes* strains with a type II-A CRISPR-Cas system, there are fewer prophages compared to type I-C CRISPR-Cas strains [22]. Interestingly, it was also shown that a unique streptococcal phage “anti-CRISPR,” a phage-encoded protein that interferes with CRISPR-Cas systems, can inhibit *S*. *pyogenes* Cas9 [23]. Future studies are needed to examine the role of anti-CRISPRs in other streptococcal species.

## Streptococcus agalactiae

*S*. *agalactiae* is a gram-positive pathobiont that colonizes the genitourinary and gastrointestinal mucosa. *S*. *agalactiae* is capable of opportunistic vertical transmission to the neonate, which can result in meningitis, chorioamnionitis, or neonatal sepsis [24,25]. Additionally, *S*. *agalactiae* is also a rare cause of infection in nonpregnant adults [26,27]. *S*. *agalactiae*, like *S*. *pyogenes*, harbors either a Type-I-C or Type-II-A CRISPR system, which has been exploited to produce CRISPRi strains like those generated in *S*. *pyogenes*. Notably, the residues associated with Cas9 catalytic activity do vary from *S*. *pyogenes*, with the HNH nuclease domain shifted such that the point mutation associated with catalytic inactivation is H845A rather than H840A [19,28].

CRISPRi has been applied as a tool for the generation of rapid, flexible knockdown phenotypes [28,29]. CRISPRi has also been utilized to investigate the various roles that the CRISPR/Cas system plays apart from its canonical role as a nuclease, highlighting a potential role in regulating virulence, which has been suggested in previous non-CRISPRi studies [28,30]. There has been a recent surge in the development of CRISPRi technologies for *S*. *agalactiae*. A group has developed multiple dCas9 strains for use in exploring *S*. *agalactiae* transcription and fitness [28,29]. These developments can be expanded on for future research, with the potential for added complexity using sgRNA multiplexing.

## Streptococcus pneumoniae

*S*. *pneumoniae* is a gram-positive pathobiont colonizing the upper respiratory mucosa that can cause pneumonia, meningitis, or otitis media [31]. *S*. *pneumoniae* does not possess an endogenous CRISPR/Cas system, but the natural competence and relative ease of genetic manipulation simplifies most CRISPRi-based applications. One common approach is the chromosomal insertion of Spy dCas9 and a single-guide RNA (sgRNA), allowing for expression. Additionally, this system can be driven by an inducible promoter, allowing for greater experimental complexity [32,33].

In *S*. *pneumoniae*, CRISPRi has been applied in combination with high-throughput Tn-seq (transposon sequencing) [34]. Tn-seq-based essential gene identification can be followed up with CRISPRi screening to identify previously unexplored genes. Tn-seq has been utilized in *S*. *pneumoniae* strain D39 for the identification of genes potentially related to antibiotic resistance [33]. Another application of CRISPRi is CRISPRi-seq and its notable subtype sCRilecs-seq. CRISPRi-seq is an approach that allows for minimal off-target effects through careful targeting of all operons rather than individual genes [32]. sCRilecs-seq (subsets of CRISPR interference libraries extracted by fluorescence-activated cell sorting coupled to next-generation sequencing) is a process that couples CRISPRi with fluorescence-activated cell sorting (FACS) to identify loci associated with a given phenotype [35]. Bacteria with desired fluorescence intensities can be collected and subject to sequencing to determine identity. This technique has been used to identify pathways that could function as combination treatment targets to deal with the growing threat of ß-lactam resistance in *S*. *pneumoniae* [36]. The ease of mutagenesis in *S*. *pneumoniae* facilitates the utilization of complex screening techniques, allowing CRISPRi technology to continue to expand and provide valuable results.

## Future directions in CRISPRi technology

CRISPRi is a growing technology, with recent developments across different bacteria. The strengths of CRISPRi motivate continued development of screening technology as rapid, targeted expression manipulation is highly versatile. The push to develop CRISPRi technology in *S*. *agalactiae* is notable, yielding insights into transcription and fitness. Additionally, advanced applications such as SCRilecs-seq and CRISPRi-seq in *S*. *pneumoniae* or the nisin-inducible system developed for *E*. *faecalis* allow for increasingly complex and specialized investigations into antibiotic resistance mechanisms and virulence associated genes [15,32].

A future direction of interest would be utilization of Mobile CRISPRi, a technique developed for *Pseudomonas aeruginosa*. Mobile-CRISPRi allows for the transfer and integration of CRISPRi into bacteria through an antibiotic resistance cassette, sgRNA spacers, and a promoter that drives dCas9 expression [37]. CRISPRi technology is expanding and continues to demonstrate exciting results as it is used in a wider span of bacterial species. Additionally, CRISPRi may be of significant value to agricultural fields requiring scalable and functional investigation into target genes [38,39].
